# Cancer survivorship in low- and middle-income countries: challenges, needs, and emerging support strategies

**DOI:** 10.3389/fpubh.2025.1601483

**Published:** 2025-07-16

**Authors:** Gustavo Rodrigues dos Anjos, Guilherme Falcao Machado, Cassiano Pereira de Barros, Victor Piana de Andrade, Rui Monteiro de Barros Maciel, Lucas Leite Cunha

**Affiliations:** ^1^Emergency and Evidence-Based Medicine Unit, Department of Medicine, Escola Paulista de Medicina, Universidade Federal de São Paulo, São Paulo, Brazil; ^2^Laboratory of Molecular and Translational Endocrinology, Department of Medicine, Escola Paulista de Medicina, Universidade Federal de São Paulo, São Paulo, Brazil; ^3^A.C.Camargo Cancer Center, São Paulo, Brazil

**Keywords:** cancer survivorship, low-and middle-income countries (LMICs), late effects, health disparities, quality of life

## Abstract

Cancer survivorship has become a critical global health issue, with survival rates on the rise in both high-income countries (HICs) and low- and middle-income countries (LMICs). Cancer survivors, encompassing individuals from diagnosis onward, face unique and complex health challenges that necessitate tailored care. In HICs, survival rates have increased due to advances in diagnosis and treatment, prompting robust survivorship programs addressing late effects and long-term quality of life. In LMICs, however, disparities in healthcare access, infrastructure, and support systems hinder comparable progress in survivorship care, particularly outside urban areas. LMIC survivors often contend with financial barriers, limited access to follow-up care, and significant psychosocial and rehabilitative gaps. Specialized survivorship centers are rare, and resources for addressing late effects are constrained, impacting survivors' long-term wellbeing. Emerging studies, primarily from middle-income nations, identify late effects such as endocrine and metabolic disorders, though robust, comprehensive data remain scarce. For childhood cancer survivors, late effects like chronic viral infections and cognitive impairments are documented, yet systematic follow-up remains limited. To bridge these gaps, LMICs require innovative care models, such as non-profit partnerships and community-based interventions, to meet the complex needs of survivors. In Brazil, we've highlighted successful programs including the *Mais Médicos* program for increased care capacity and DATA-SUS as a model registry. This review synthesizes available literature on cancer survivorship in LMICs, evaluating challenges and successful practices across diverse regions. Addressing these needs is crucial for improving survivorship care, particularly in regions where socioeconomic disparities amplify the challenges of post-cancer recovery.

## Introduction

Cancer survivors—those living with cancer from diagnosis onward—represent a growing population (1). A cancer diagnosis marks a turning point in an individual's life, introducing health and social needs that require focused care (2). In high-income countries, 5-year survival rates have reached nearly 50%, thanks to advancements in therapy and early diagnostics, with even higher rates in pediatric and young adult cancers (1).

In low- and middle-income countries (LMICs), cancer survivorship presents additional challenges, such as limited oncologic care, early diagnostics, and effective treatments (3). Survival rates are generally lower in LMICs due to disparities in infrastructure, professional training, and financial barriers (4). Survivors in LMICs also face insufficient support, particularly in follow-up programs, rehabilitation, and psychosocial care—critical components of quality survivorship (5). This review examines cancer survivorship in LMICs, highlighting the challenges and successful strategies that can be adapted to meet local needs. This narrative review was conducted in accordance with the SANRA (Scale for the Assessment of Narrative Review Articles) guidelines, which provide a framework to ensure clarity, justification of the topic, scientific reasoning, appropriate referencing, and relevance to the field (6). Although this study is presented as a narrative review, we adopted a semi-structured and purposeful approach to identify relevant literature. We performed a comprehensive search of peer-reviewed publications across major databases, including PubMed, Scopus, and Web of Science, focusing on literature published between 2000 and 2024. Our search strategy combined terms related to “cancer survivorship,” “low- and middle-income countries,” “late effects,” and “health disparities.” Priority was given to studies that provided empirical data, cohort analyses, or policy insights relevant to LMIC contexts. We also included gray literature, such as reports from international health organizations, to capture region-specific initiatives and challenges not yet represented in indexed literature. We prioritized large cohort studies, global reports, and conceptual analyses relevant to LMICs, ultimately selecting approximately 90 sources based on thematic relevance, geographic diversity, and scientific rigor. This approach enabled us to integrate empirical findings with broader policy and systems-level considerations, in line with accepted principles of narrative synthesis in health sciences. While formal inclusion and exclusion criteria were not rigidly applied, we ensured thematic relevance, geographical diversity, and methodological quality in the selection process. The narrative review format was intentionally chosen to allow flexibility in synthesizing diverse sources and to accommodate the heterogeneity of survivorship models and health systems across LMICs.

While previous reviews have largely centered on survivorship in HICs, this review offers a novel and timely contribution by focusing specifically on LMICs—settings where the cancer burden is rising but survivorship remains a neglected area of study and policy. This review not only highlights gaps in survivorship data and service delivery in LMICs but also proposes strategies to inform future policy and practice.

## A brief overview of cancer survivorship

Cancer survivors require more than clinical care. Their needs include social, psychological, and economic support, as they may face stigma, workforce reintegration issues, and barriers to daily activities. Long-term complications, such as cardiovascular disease, metabolic dysfunction, and mental health issues, are often under-addressed. Survivorship challenges are grouped into several categories according to the affected system as can be seen in Table 1.

**Table 1:** Main clinical and surgical sequelae of cancer survivors.

**Clinical survivorship**	**Therapies involved**	**Main conditions**
Cardiovascular survivorship	Radiotherapy (exposing the chest), Anthracyclines, alkylating agents (eg. cisplatin), biological therapies and immunotherapies (eg. tyrosine kinase inhibitors, immune checkpoint inhibitors) and hormonal therapies (GnRH agonists)	Heart failure, coronary artery disease, atrial fibrillation, lymphedema
Pulmonary survivorship	Radiotherapy, bleomycin, alkylating agents (busulfan), hematopoietic stem cell transplant, thoracic surgery, immunotherapies and antibody drug conjugates	Pulmonary fibrosis, interstitial pneumonitis, restrictive lung disease, Superimposed infections, Pulmonary dysfunction and pulmonary toxicity
Endocrine survivorship	Cranial, abdominal and neck radiotherapy, alkylating agents, heavy metals, antimetabolites, immunotherapy, tyrosine kinase inhibitors, cranial and endocrine surgeries	Hypothyroidism, precocious puberty, adrenal insufficiency, GH deficiency, hypogonadism, gonadal failure, thyroid cancer, metabolic syndrome/diabetes/dyslipidemia, hypocalcemia/vitamin D, osteoporosis
Ophthalmological survivorship	Radiotherapy, cranial surgeries, chemotherapy	Dry eyes, double vision, cataracts, blurred vision, retinopathy and glaucoma
Otological survivorship	Radiotherapy, platinum-based chemotherapy, surgery, aminoglycosides and loop diuretics	Hearing loss, tinnitus.
Neurological survivorship	Chemotherapy, radiotherapy directed at the CNS	Cognitive impairment, chronic pain, peripheral neuropathy
Hematological and immune survivorship	Chemotherapy, hematopoietic stem cell transplantation	Anemia, immune deficiency
Gastrointestinal survivorship	Radiotherapy, chemotherapy, surgical interventions	Micronutrient deficiency, anemia, diarrhea, nausea and vomiting, constipation, bloating and abdominal pain.
Renal survivorship	Chemotherapy (ifosfamide, methotrexate, cisplatin), abdominal radiotherapy, surgical interventions (nephrectomies)	Decreased eGFR, proteinuria, tubular dysfunction, hypomagnesemia, hypertension, hypophosphatemia.
Musculoskeletal survivorship	Radiotherapy with high doses, chemotherapy (ifosfamide, L-asparaginase, methotrexate, vincristine), glucocorticoid therapy, bisphosphonate therapy, denosumab, bone surgeries	Muscular atrophy, fibrosis and hypoplasia. Spinal malalignment, osteonecrosis, chest wall/cranial/orbital deformities, limb length discrepancy, bone hypoplasia and spinal growth retardation,
Psychiatric and mental health survivorship	Radiotherapy, chemotherapy and surgery	Mood disorders, post-traumatic stress disorder.

### Cardiovascular survivorship

Survivors are at higher risk for heart failure (HF), coronary artery disease (CAD), and atrial fibrillation (AF). These risks depend on cancer type, treatment, genetics, inflammation, and common risk factors. Specific cancers linked to CAD include esophageal adenocarcinoma, lung cancer, and hematologic cancers (7). Radiotherapy, particularly for Hodgkin's lymphoma and breast cancer, increases CAD and HF risk (8, 9). Pediatric cancer survivors face a 15-fold higher risk of HF than their peers (9). Chemotherapies like anthracyclines and cisplatin contribute to cardiovascular complications, with cumulative anthracycline doses increasing HF risk (10). Lymphedema, especially in breast cancer survivors, can significantly affect quality of life (11).

### Respiratory survivorship

Cancer treatments can cause lung damage, leading to symptoms like dyspnea and reduced exercise capacity (12). Post-treatment issues such as pulmonary fibrosis, interstitial pneumonitis, and restrictive lung diseases are common, especially in lung cancer survivors. Radiation to the chest increases risks of lung fibrosis and chronic pneumonia. Chemotherapies like bleomycin and alkylating agents also contribute to lung damage. Hematopoietic stem cell transplantation (HSCT) can cause severe pulmonary complications due to chemotherapy, radiotherapy, and immune reactions.

### Endocrine survivorship

Endocrine sequelae from cancer treatments are diverse, including issues with the hypothalamic-pituitary axis, gonads, thyroid, bone density, and obesity. Radiotherapy, especially cranial, can cause growth hormone (GH) deficiency and early puberty. Chemotherapy and radiotherapy are linked to gonadal failure, and HSCT patients often experience hypogonadism, hypothyroidism, and GH deficiency. Treatment with immune checkpoint inhibitors can lead to hypothyroidism and type-1 diabetes (13). Diabetes risk increases, likely due to insulin resistance, obesity, and chemotherapy (14).

### Ophthalmological and otological survivorship

Ocular side effects of cancer treatment include dry eyes, double vision, cataracts, and retinopathy. Radiation to areas near the eyes increases the risk of these effects, particularly in CNS cancer and leukemia survivors. Otological side effects, like hearing loss and tinnitus, result from radiation or chemotherapy (15). These conditions can be particularly challenging in children, affecting their language and communication development (16).

### Neurological survivorship

Cognitive impairment, including memory and learning difficulties, is common in cancer survivors, particularly those treated with methotrexate or brain radiation (17). Peripheral neuropathy, often linked to chemotherapy, can persist as chronic pain syndrome (18). Chronic pain, a prevalent symptom, is associated with reduced quality of life and should be adequately managed (19).

### Hematological and immunological survivorship

Anemia, common in cancer survivors, contributes to fatigue and other symptoms. Chemotherapy affects immune responses, increasing the risk of infections, especially in HSCT recipients (20).

### Gastrointestinal survivorship

GI symptoms are frequent in survivors of colon and rectal cancers, often persisting after chemotherapy and radiation. Symptoms such as constipation, diarrhea, and abdominal pain are common but underreported (21). Chemotherapy disrupts gut microbiota, leading to inflammation and symptoms that affect psychosocial health. Upper GI cancer patients often suffer from altered gastrointestinal function, compromising their nutritional status (22).

### Renal survivorship

Chronic kidney disease is common among cancer survivors, especially those who underwent abdominal radiation or chemotherapy (23). Nephrectomy patients also need long-term monitoring for kidney function (24).

### Musculoskeletal survivorship

Radiotherapy causes long-term musculoskeletal effects, including muscular atrophy, bone malformations, and growth retardation. Chemotherapies like methotrexate and vincristine, combined with radiation, exacerbate these issues (25). Osteonecrosis can also develop, particularly in older patients or those receiving glucocorticoid therapy post-HSCT (26).

### Psychiatric and mental health survivorship

Cancer survivors experience higher rates of mood and anxiety disorders. Depression and anxiety are prevalent in both pediatric and adult survivors, with children facing an increased risk of post-traumatic stress disorder (27). Female sex and older age are associated with worse mental health outcomes.

### Social and economic impacts of cancer survivorship

Cancer survivors often face unemployment, with those diagnosed with childhood cancer being particularly affected (28). Cancer treatments can impair work productivity and reduce income (29). Social isolation is also a significant issue, negatively impacting psychological wellbeing, particularly in pediatric survivors (30, 31).

## Overview of cancer in LMICs

**Figure 1:**
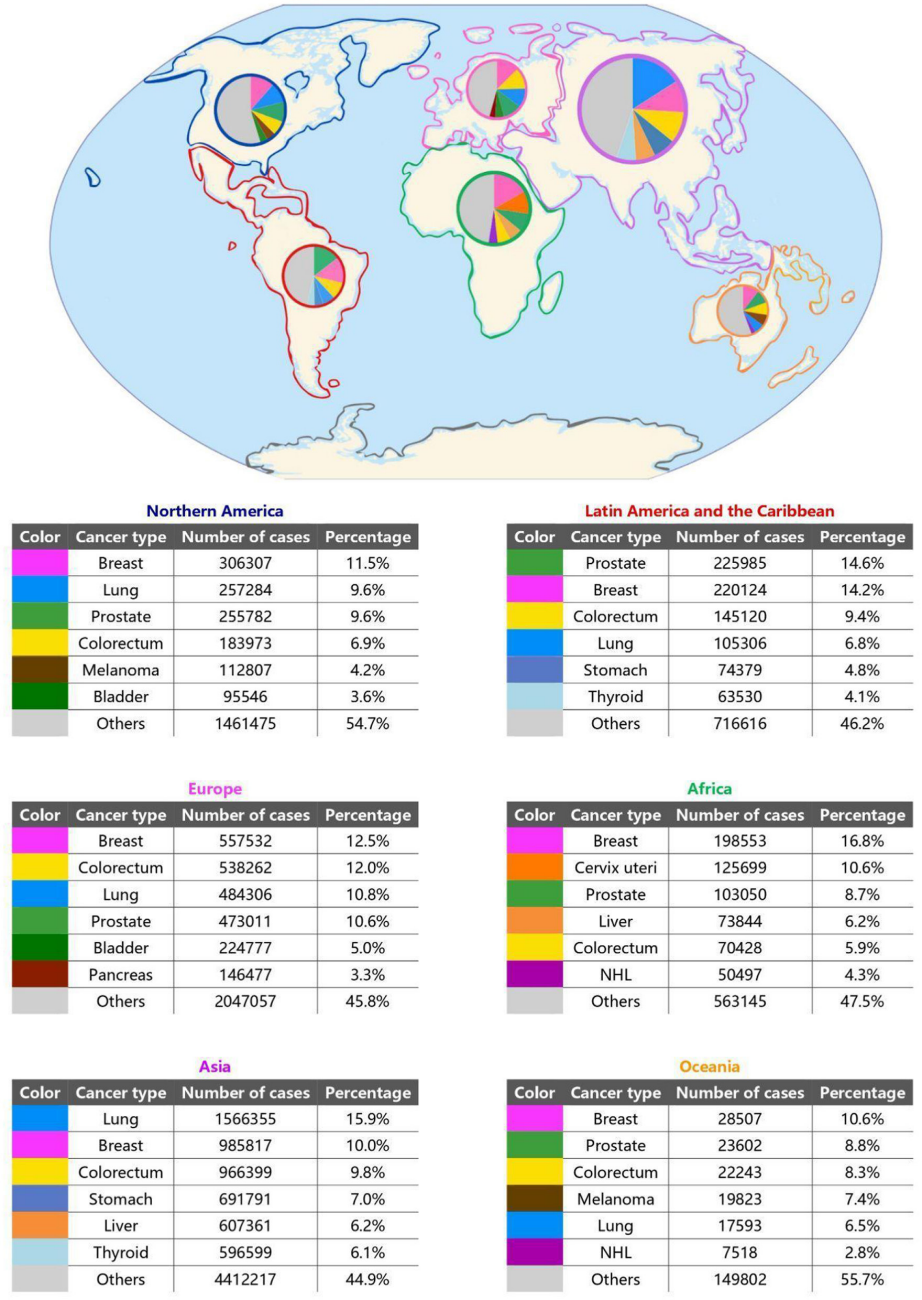
Global map with pie-charts presenting the most incident types of cancer by region (78–83). It's important to note that regions with higher Human Development Index (HDI) and high-income countries (HIC), such as Northern America, Europe and Oceania, have a predominance of lifestyle related malignancies (lung and colorectum cancer, for example); whereas regions with lower HDI and low- and middle-income countries (LMIC), such as Africa, Asia, Latin America and the Caribbean, have more infection-related malignancies (cervix uteri and liver cancer, in particular).

Cancer has become an increasingly significant public health issue not only in high-income countries (HICs) but also in low- and middle-income countries (LMICs). Figure 1 shows the regional distribution of cancer types based on income levels. Countries with high Human Development Index (HDI) such as North America and Western Europe have higher cancer incidence rates, with improved survival outcomes due to better healthcare infrastructure and treatment. In contrast, regions with lower HDI like Latin America, Africa, Asia, and Eastern Europe tend to experience higher age-standardized mortality rates despite having lower crude incidence rates. This suggests that treatment effectiveness is lower, leading to poorer survival outcomes.

In Brazil, for example, cancer mortality rates have been surpassing cardiovascular disease mortality, reflecting a broader trend in many LMICs undergoing epidemiological transitions (32). Data from Brazil's 5,570 municipalities from 2000 to 2019 reveal that cancer mortality increased in over half of the states, particularly in higher-income areas. This shift emphasizes the rising importance of cancer in LMICs and the urgent need for targeted public health responses.

Globally, disparities in cancer outcomes between HICs and LMICs are striking. While HICs benefit from advanced diagnostic technologies, treatment options, and healthcare investments, LMICs struggle with systemic gaps that hinder cancer control, resulting in worse outcomes (33, 34). These disparities highlight the need for tailored approaches in LMICs to address their unique challenges, including limited resources and lack of specialized care.

Reliable cancer data collection is essential for effective public health strategies; however, many LMICs lack comprehensive cancer registries (35). Brazil's DATA SUS is an example of an established data collection system, but most LMICs still face gaps in data, hindering the development of targeted prevention and treatment programs. In contrast, HICs have robust cancer registries that support early detection and inform policy (36).

Delays in cancer diagnosis are common in LMICs, where patients often present with advanced-stage cancer due to limited access to diagnostic services and low awareness of cancer symptoms (37). Screening programs for breast, cervical, and colorectal cancers are often unavailable or inaccessible, and existing interventions lack clinically relevant measures (38, 39). Addressing these gaps is crucial to improve cancer control and patient outcomes.

Access to cancer treatment is another significant challenge in LMICs, where oncologic care is typically concentrated in urban centers, forcing rural patients to travel long distances and incur additional costs (40). Treatment facilities in LMICs often face staff shortages and inadequate resources (41). Even in HICs, geographical and financial barriers exist, leading to disparities in treatment access and quality (42).

Limited access to first-line cancer drugs is another barrier in LMICs, where the cost of medications, especially targeted therapies, is often prohibitive (43). Although efforts to increase access to generic drugs have been successful in HICs, LMICs continue to struggle with affordability and availability (44).

Additionally, social support systems in LMICs are often insufficient, leaving patients without financial, emotional, or logistical assistance during treatment (45). In contrast, HICs generally have more robust support networks, though inequalities remain, especially in private healthcare systems (46).

Addressing these challenges will require comprehensive strategies to improve health infrastructures, early detection, access to treatment, and social support for cancer patients in LMICs. Strengthening international collaborations and policies is essential to reduce the disparities in cancer outcomes between LMICs and HICs.

### Particularities of cancer survivorship in low- and middle-income countries

Despite the growing body of literature on cancer survivorship in high-income countries (HICs), there remains a significant lack of data on late effects among cancer survivors in LMICs, where the cancer burden is substantial but long-term outcomes are largely unexplored (47). Research in LMICs is primarily focused on childhood cancer survivorship, with studies mostly originating from middle-income nations like India. Common late effects include secondary cancers, endocrine dysfunctions, reproductive issues, and cardiovascular problems, although their prevalence varies widely (5). Due to limited and heterogeneous study designs with small sample sizes, major gaps persist in understanding the types and risks of late effects in these regions, underscoring the need for systematic, comprehensive data on cancer survivorship in LMICs (5).

In LMICs, specialized centers for the long-term care of cancer survivors are scarce. However, some centers have conducted studies to shed light on this issue. For example, in India, one study examined 3,067 childhood cancer survivors and found that approximately two-thirds of the survivors experienced no late effects or only mild ones, while 15.6%, 16.2%, and 5.3% had grade 2, 3, and 4 late effects, respectively (89). Common late effects included chronic viral hepatitis (7.8%), thyroid dysfunction (7.5%), and other endocrine issues (13.6%). Notably, the incidence and severity of late effects have decreased over time, suggesting improvements in care and less aggressive treatments.

Another study of 300 survivors over 5 years identified that 23% had minimal disabilities, while 13% had moderate conditions, including cardiac problems and hypothyroidism (48). A comprehensive review of childhood cancer survivorship in India highlighted the prevalence of hepatitis B and C infections among survivors and the variability in research methodologies, making generalizations challenging (49). While many survivors report a good quality of life, there is an urgent need for monitoring strategies to detect late effects, particularly secondary cancers.

In Brazil, the Department of Pediatrics at the ACCamargo Cancer Center in São Paulo established a multidisciplinary team in 1999 to monitor long-term childhood cancer survivors (50). This team, consisting of oncologists, endocrinologists, cardiologists, and other specialists, found that 50% of patients had grade I late effects, while 22.5% had grade II, 35% had grade III, and 0.3% had grade IV. Among patients assessed for cardiac, gonadal, neurological, and renal function, a small percentage exhibited dysfunctions, highlighting the importance of multidisciplinary care for survivors.

Despite the progress made in LMICs, clinical characteristics of cancer survivors in these countries often mirror those seen in HICs, although social and financial disparities may exist. Metabolic disorders and endocrine dysfunctions are prevalent in both LMICs and HICs. In India, a study found high rates of dyslipidemia (61.8%), obesity (33%), and metabolic syndrome (12.2%) among children who had completed at least 2 years of cancer treatment (51). These findings are significant, as the coexistence of obesity and undernutrition in these children may create a unique metabolic profile. Another study indicated that childhood survivors of acute lymphoblastic leukemia (ALL) were more likely to be overweight or obese (30.8%), and had higher rates of hypertension, hypertriglyceridemia, and insulin resistance (52). Similarly, a study from Egypt found that childhood survivors of ALL had higher body mass indices and worse liver function markers than controls (53). In Brazil, children transplanted for acute leukemia, particularly those treated with total body irradiation, experienced a high prevalence of endocrinological late effects (54).

While endocrine and metabolic late effects are more common among cancer survivors, cardiovascular injuries remain the most lethal. A study in Mexico found that childhood cancer survivors treated with anthracyclines showed early signs of myocardial dysfunction, despite normal ejection fractions, and those treated with mediastinal radiotherapy were at risk for arrhythmias (55). In Brazil, studies report that approximately 10% of childhood cancer survivors in São Paulo experience late cardiovascular effects, with most being mild (56).

The current literature on cancer survivorship in LMICs highlights significant gaps in understanding compared to HICs, despite the high cancer burden in these regions. Studies on childhood cancer survivors reveal a variety of late effects that profoundly impact clinical management, such as chronic health issues. Data from urban and central areas suggest similar epidemiological trends in the late effects of cancer treatments. Therefore, there is a pressing need for innovative care models and targeted monitoring strategies to meet the unique needs of cancer survivors in LMICs. Addressing the systemic barriers to cancer survivorship care in these regions is crucial to improving outcomes for survivors.

From a global perspective, strengthening survivorship care in LMICs also aligns with WHO's broader strategic objectives in cancer control and non-communicable disease (NCD) management, which emphasize the continuity of care, integration into primary health systems, and the use of culturally relevant approaches (57).

Knowledge gaps in LMICs disproportionately affect certain populations. Gender-specific survivorship challenges—such as fertility preservation, early menopause, and the psychosocial burden of caregiving roles—are often under-addressed in LMICs, despite their substantial impact on quality of life, particularly for female survivors (58). Moreover, while survivorship data in LMICs predominantly focus on pediatric populations, limited attention has been given to the long-term needs of adult survivors, whose late effects and reintegration pathways often differ markedly. The transition from pediatric to adult care further complicates this landscape and remains a critical yet underexplored area (59).

## Challenges and opportunities in cancer survivorship in LMICS

Cancer survivorship in LMICs faces significant challenges, including limited healthcare infrastructure, financial barriers, and psychological stigma. A 2022 survey by the Survivorship Special Interest Group of the International Psycho-Oncology Society highlighted that services such as reproductive health, genetic counseling, and distress management were more readily available in HICs compared to LMICs, with major barriers in LMICs including a focus on treatment rather than survivorship (60). Furthermore, a shortage of trained oncology professionals limits comprehensive survivorship care, particularly in rural areas, where survivors face high out-of-pocket costs and long travel distances to access care (61).

The social stigma surrounding cancer in LMICs exacerbates these challenges, leading to difficulties in survivors' relationships and limiting their access to mental health support (62). Survivors often face economic hardship due to direct treatment costs and indirect financial impacts, such as unemployment and discrimination. A Brazilian study revealed that 38% of survivors of childhood cancer still depended on their parents financially, while others faced social challenges, including smoking and drug use (50). These issues are particularly pronounced in LMICs, where financial instability and social vulnerability add to the burden.

Additionally, the availability of mental health services is limited, leaving many survivors without adequate psychological support. In Brazil, over a third of childhood cancer survivors reported cognitive impairments or pain, and a quarter had emotional difficulties (60). A study in Malaysia found that more than half of childhood cancer survivors experienced moderate or higher levels of anxiety, with diagnosis at advanced stages being a key predictor of poor health-related quality of life (63). These findings underscore the need for mental health resources and improved quality of life assessments in LMICs.

A major concern in cancer survivorship care is the identification and early diagnosis of secondary malignancies. Survivors of retinoblastoma, for example, are at an elevated risk of developing secondary cancers due to treatment. A study in Argentina found that 3.36% of retinoblastoma survivors developed secondary tumors, with radiation increasing the risk of certain cancers, such as Ewing sarcoma, by 700 times in hereditary survivors (64). This highlights the need for ongoing surveillance, genetic counseling, and early detection strategies for survivors at risk of secondary malignancies.

Improving cancer survivor care in LMICs requires strengthening community-based care models, like Brazil's Mais Médicos and Family Health Strategy, which bring healthcare professionals to underserved populations, fostering integrated care (65). Empowering local health workers and primary care providers to manage survivorship needs can ease the burden on oncology centers and improve care accessibility. Expanding telemedicine also offers a viable solution for remote symptom management, reducing the travel burden on survivors in rural areas (66).

Traditional and complementary medicine (T&CM) remains a key component of healthcare in many low- and middle-income countries (LMICs), where it often represents the most accessible or sole form of care, as noted by the World Health Organization (WHO) (67). However, cancer survivorship strategies seldom incorporate integrative approaches. Therapies such as mind-body interventions, phytotherapy, acupuncture, and nutritional support have demonstrated potential in managing common survivorship symptoms, including fatigue, anxiety, chronic pain, and gastrointestinal issues. While rigorous evaluation and regulation are necessary, integrating culturally relevant, evidence-informed T&CM into survivorship care may offer scalable, cost-effective solutions in resource-limited settings. Incorporating traditional healing within community care, primary care training, and policy frameworks could improve cultural acceptability, symptom management, and service accessibility. This approach supports WHO's recommendation to evaluate and integrate traditional medicine into health systems as part of universal health coverage efforts (68).

**Figure 2:**
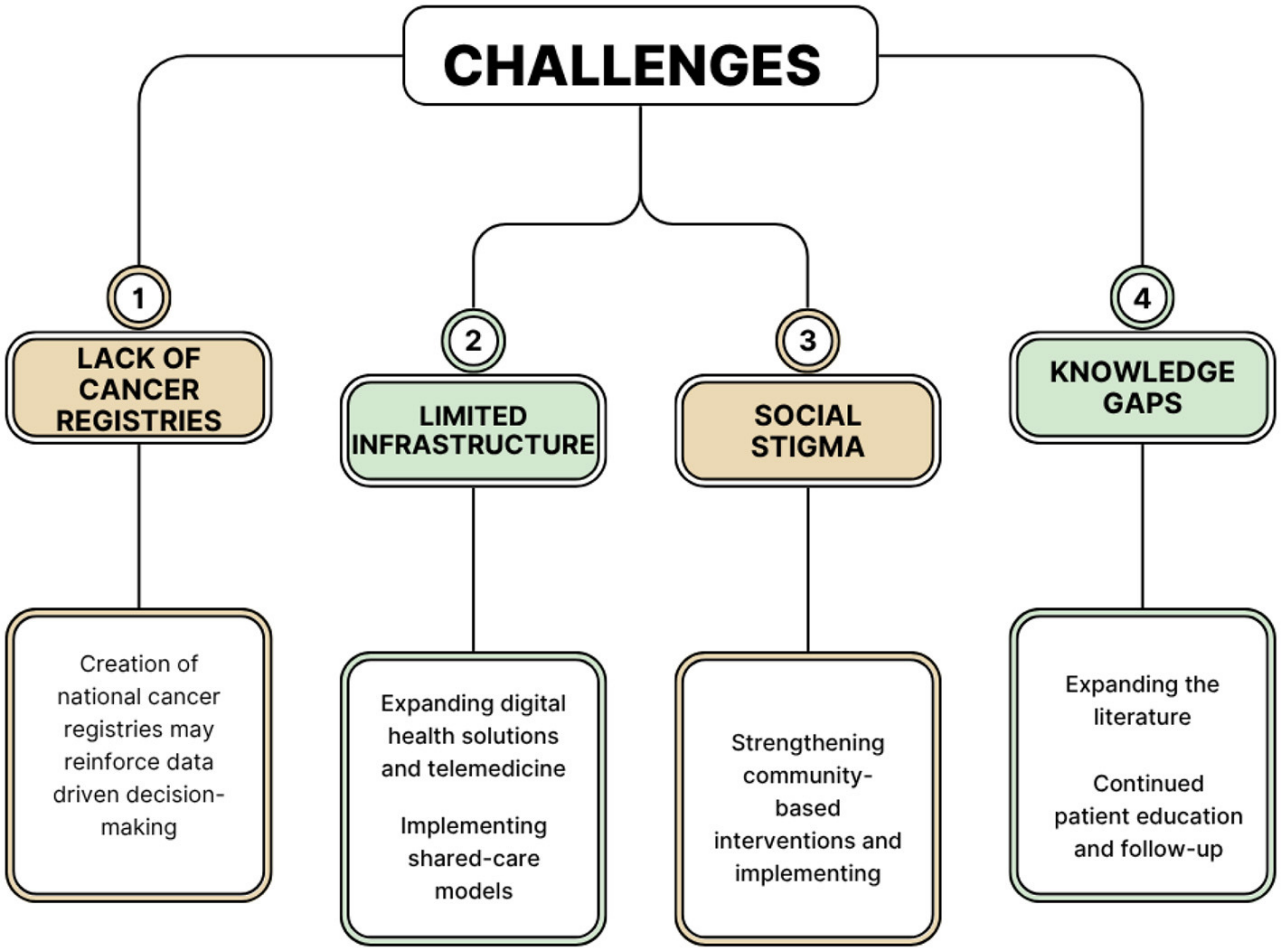
A conceptual diagram which summarizes the most important Challenges faced by cancer survivors in LMICs and the possible interventions that may improve care provided for patients.

International collaborations and non-governmental organization support are crucial in building sustainable cancer survivorship programs in LMICs (69). These partnerships can provide essential resources, expertise, and infrastructure, enhancing care delivery. Task-shifting and shared-care models, where general practitioners manage routine follow-up care, can further decentralize survivorship care and improve patient-centered outcomes (70). Integrating survivorship care into national health policies and advocating for policy changes are also critical to ensuring long-term sustainability and adequate resources for cancer survivors (71). We have highlighted many opportunities for the various challenges faced by LMICs and cancer survivors in LMICs, as well as experiences worldwide, in a conceptual framework as seen in Figure 2.

Lastly, the development of comprehensive cancer registries can help identify gaps in care and inform region-specific interventions (72). By collecting detailed data on survivor outcomes, these registries can support evidence-based policies and improve care coordination, ensuring better long-term outcomes for cancer survivors.

By integrating findings from diverse geographic regions and levels of healthcare development, this review proposes a contextual framework for survivorship care in LMICs that includes community-based strategies, non-profit engagement, and scalable care models tailored to resource-limited settings. Moreover, this review proposes a contextualized framework for survivorship care in LMICs, emphasizing integrated primary care, local health worker engagement, and the inclusion of traditional medicine as potential tools for expanding survivorship support in low-resource settings. These insights provide a foundation for future research and policy efforts aimed at reducing global disparities in cancer survivorship. A summary of challenges and opportunities faced by LMICs can be seen in Table 2.

**Table 2:** Challenges and opportunities of the patient centered cancer survivor units in LMICs.

**Challenges and needs**	**Opportunities**	**Similar experiences**
Lack of cancer registries	Creation of national cancer registries may reinforce data driven decision making	DATA-SUS, Brazil. DATA-SUS is Brazil's public health data system, managed by the Ministry of Health. It collects, processes, and provides access to health-related data, covering everything from hospital admissions, epidemiological information, mortality rates, and public healthcare infrastructure.
Limited healthcare infrastructure in rural and underserved areas	Expanding digital health solutions and telemedicine	West China Hospital of Sichuan University, China. The West China Hospital of Sichuan hosted a network connecting 249 hospitals in 112 rural cities with highly specialized urban centers, from 2002 to 2013. Telehealth projects in India: Apollo, OTRI, Asia Heart Foundation Egyptian Telemedicine Network, Egypt (84)
Psychological and social stigma: loss of income and employment	Strengthening patient organizations, local community support and legislation, empowering patients	The Universal Health Care Act and the National Integrated Cancer Control Act in the Philippines in 2019 were able to be passed due to the efforts of a country-wide coalition of Filipino patient organizations.
Poor availability of mental health resources	Strengthening community-based interventions	Blossom Program (85), Saudi Arabia. The Blossom Program was a support group on psychological distress and quality of life in breast cancer patients in Saudi Arabia, which showed statistically significant increases in overall quality of life and decreases in anxiety and depression scores.
Shortage of trained professionals (for oncology, cardiovascular, endocrine, reproductive, and other survivorship)	Implementing shared-care models, training family medicine doctors to handle routine follow-up	A study evaluating a shared-care model for prostate cancer in Australia (86) showed no clinically important statistically significant differences between the groups. Furthermore, shared care was more cost-effective and preferred by the patients versus the usual hospital care.
Lack of cancer survivorship research and knowledge gaps	Expanding the literature with research that details disease burden and outcomes.	A Survivorship Research in Prostate Cancer (SuRECaP) (87) working group, composed of researchers and clinicians interested in prostate cancer survivorship was formed in order to improve the quality of prostate cancer survivorship research.
Low awareness among patients and their relatives about the needs of cancer survivors	Continued patient education and follow-up.	Various modalities of educational intervention have been proposed, and the results appear to reduce anxiety, depression, psychological distress and pain (88).

## Comparison of cancer survivorship in HICS and LMICS

High-income countries (HICs) have made significant strides in managing the long-term sequelae of cancer and its treatment through the development of structured survivorship care frameworks. These include the implementation of survivorship care plans, multidisciplinary follow-up programs, and risk-based stratification models that allow for tailored surveillance and early intervention (73, 74). For instance, late effects such as cardiovascular disease, endocrine disorders, and cognitive impairment are routinely monitored in childhood and adult cancer survivors through well-integrated care pathways supported by national guidelines and electronic health records (75). Furthermore, the robust systems and care pathways present in HICs are often more cost-effective (73) than unstructured care, which is often present in LMICs.

In contrast, LMICs face persistent challenges in addressing these late effects due to fragmented health systems, limited access to specialized care, and the absence of standardized follow-up protocols. Some LMICs have initiated promising survivorship strategies. For example, survivorship clinics in Brazil have begun implementing follow-up protocols inspired by HIC guidelines but adapted to local resource constraints, often relying on nurse-led care or telemedicine platforms as alternatives (76). In India, the Indian Cancer Society's Project PICASSO (Partnership in Cancer Survivorship Optimization) helps to implement hospital-based survivorship clinics with success (77).

By comparing the structured, evidence-based models of survivorship care in HICs with the emerging, context-sensitive interventions in LMICs, it becomes evident that scalable and sustainable survivorship solutions require both innovation and policy commitment. Strategies such as task-shifting, mobile health interventions, and regional centers of excellence could bridge current gaps, particularly if supported by international collaborations and health system strengthening. Drawing from HIC experiences, LMICs have the opportunity to adapt survivorship frameworks in ways that are feasible, culturally appropriate, and equity-driven.

## Limitations

This narrative review offers a broad synthesis of cancer survivorship in low- and middle-income countries (LMICs), yet several limitations must be acknowledged. First, the semi-structured and purposive literature search, while comprehensive, does not adhere to systematic review protocols, which may introduce selection bias. Without formal inclusion and exclusion criteria, there is a risk of omitting relevant studies, particularly those published in languages other than English or indexed in non-mainstream databases.

Additionally, the heterogeneity of the included studies, in terms of design, quality, and outcome measures, limits the ability to draw definitive conclusions or perform meta-analytic comparisons. Much of the existing research from LMICs focuses on pediatric populations, leaving a significant gap in data regarding adult cancer survivors, especially in rural and marginalized communities. Furthermore, survivorship studies from LMICs are often hospital-based and may not reflect the broader population of survivors who do not receive follow-up care or whose outcomes remain undocumented.

Lastly, survivorship experiences and health system responses are deeply influenced by sociocultural, economic, and political contexts that may not be fully captured in the existing literature. The absence of longitudinal studies and national cancer registries in many LMICs hinders efforts to assess long-term outcomes and the effectiveness of interventions.

## Closing remarks

In conclusion, enhancing cancer survivorship care in LMICs requires tailored strategies that address specific challenges. Key approaches include conducting research to identify survivor needs, implementing community-based interventions, and advocating for policy changes to ensure equitable access to healthcare services. Collaborating with local organizations and stakeholders is essential for creating a supportive network that promotes the well-being of survivors. By prioritizing these strategies, we can improve healthcare systems and the quality of life for cancer survivors in LMICs. In this review, we offer novel contributions by centering the lived realities of cancer survivors in LMICs, a population often underrepresented in the global survivorship discourse. Unlike prior reviews that tend to generalize survivorship experiences or focus predominantly on high-income settings, our synthesis highlights region-specific challenges such as limited follow-up infrastructure, sociocultural barriers to care, and gaps in psychosocial support. Furthermore, we identify emerging patterns of late effects—particularly endocrine and metabolic disorders—in LMIC contexts and draw attention to the lack of systematic data collection and survivorship tracking. Future research should prioritize longitudinal investigations into the survivorship landscape within low- and middle-income countries (LMICs), with particular attention to the integration of survivors' experiences and findings. Additionally, efforts should be directed toward the design and evaluation of scalable, context-specific care models. Collaborative platforms that facilitate data exchange between countries could also be evaluated to assess how well they support innovation in the care of survivors.
